# Nurses’ Clinical Work Experience during Pregnancy

**DOI:** 10.3390/healthcare9010016

**Published:** 2020-12-24

**Authors:** Hyunjung Lee, Hyoung Eun Chang, Jiyeon Ha

**Affiliations:** College of Nursing, Konyang University, Daejeon 35365, Korea; leehj18@konyang.ac.kr (H.L.); heather_chang@konyang.ac.kr (H.E.C.)

**Keywords:** nurse, pregnancy, shift-work, qualitative research

## Abstract

The working environment of nurses contains numerous hazards that can be particularly harmful to pregnant women. In addition, pregnancy-induced changes can themselves cause discomfort. Therefore, it is necessary to analyze pregnant nurses’ experiences of clinical work. This qualitative study analyzed data collected through in-depth interviews. From January to June in 2020, 12 shift-work nurses who had experienced pregnancy within three years were interviewed. The main question was “Could you describe your clinical work experience during pregnancy?” Qualitative data from field notes and transcriptions of the interviews were analyzed using Colaizzi’s method. Six categories were extracted that described the nurses’ clinical work experience during pregnancy, as follows: “enduring alone,” “organizational characteristics of nursing,” “risky work environment,” “strengths that sustain work during pregnancy,” “growth as a nurse,” and “methods to protect pregnant nurses.” Pregnant nurses experienced various difficulties due to physical and mental changes during pregnancy, and the clinical working environment did not provide them with adequate support. The findings of this study will be helpful for developing and implementing practical maternity protection policies and work guidelines.

## 1. Introduction

According to statistics published by the Korean Nurses Association [1], although the proportion of male nurses is increasing, more than 96% of nurses in Korea are women. Moreover, since nurses working in hospitals tend to be in their 20s to 40s, corresponding to their reproductive years, regularly monitoring of maternity protection in the nurses’ workplace is necessary. Previous research found that 33.5% of hospital nurses continued to work night shifts after becoming pregnant, 28.5% experienced “taking turns” getting pregnant, and 23.8% experienced miscarriage or stillbirth while at work [2].

In South Korea, a clause in the Labor Standards Act revised in 2010 stipulates that pregnant women cannot work night shifts or weekend shifts, but if pregnant women request explicitly, it is possible for them to work nights and weekends upon approval of the Ministry of Employment and Labor [3]. According to a new protection clause for pregnant women added in 2014, if women pregnant for less than 12 weeks or more than 36 weeks request their work hours to be reduced by 2 h a day, this request should be granted, and the daily work hours of workers who work less than 8 h could be reduced to 6 h per day [4]. However, despite the Labor Standards Act, 34.1% of health care workers replied that they did not feel free to get pregnant in 2018 [5]. The proportion of pregnant nurses with reduced work hours was 11.4%, and 16.6% worked night shifts [5].

All 18 nurses who participated in a recent study worked shifts while they were pregnant, and four of those 18 nurses experienced miscarriage while at work [6]. Kim and Lee [7] reported that the fatigue, stress, and burnout of pregnant nurses were equivalent to those of patients with chronic illnesses, even for nurses who did not work shifts. Engaging in clinical work while pregnant is already associated with high fatigue, stress, and burnout among nurses; therefore, shift-work causes the physical condition of pregnant nurses to deteriorate due to constantly changing rhythms, they feel guilty towards their colleagues and their unborn babies, and they seriously consider quitting [6]. The characteristics of nursing work such as time pressure, physical and emotional labor, and shift-work have been found to increase nurses’ intention to quit regardless of pregnancy [8]. Therefore, carrying out a normal nursing work load while pregnant can be a major burden, both physically and mentally.

According to previous researchers, health care workers are exposed to a variety of biological, chemical, and physical risks [9]. Nurses have a particularly high exposure to these risks [10], especially infectious diseases [11], radiation [12], and chemotherapy [13]. In fact, nurses engaged in clinical work have higher rates of premature birth, miscarriage, and delivery by cesarean section than women in other professions [14,15]. More than 46% of pregnant nurses experience complications such as vaginal bleeding, and the risk of complications increases among nurses who are required to remain constantly on their feet during clinical work or to work overtime [16]. Previous studies have confirmed that the nurses’ work environment is highly dangerous for pregnant women, and this issue applies both to South Korea [17] and to other countries [11,13,15].

For women to work and maintain a family life in a balanced way, issues regarding marriage, birth, and childcare are very important. Without consideration and support from their workplace, women can be forced into leaving the workforce. Since nurses face particularly serious exposure to various risk factors in their work environment, it is especially important for the workplace to provide a safe environment for pregnant nurses and their fetuses, including support to reduce stress. Although 10 years have passed since South Korea’s Labor Standards Act has been revised, problems appear to persist, as shown in many research studies and reports, and these issues are becoming lower priorities for clinicians and managers.

A study that investigated methods to protect pregnant health care workers suggested that a health management nurse at the medical institution should provide health management and check on the condition of pregnant women, management should protect pregnant women’s right to avoid risky tasks, and the administration should prepare procedures and guidelines that can protect pregnant women [18]. Throughout the world, nurses are required to engage in clinical work, with its accompanying risk factors, during pregnancy; this fact underscores the need for protective measures. For this reason, researchers should focus on the issues facing pregnant nurses and conduct studies to identify and improve risks and problems in the field

This study aimed to obtain insights into the meaning of nurses’ clinical work during pregnancy and to investigate the essence of those experiences by conducting in-depth interviews about the clinical work experiences of nurses who were on a three-shift schedule during their pregnancy and analyzing the data using qualitative research methods.

## 2. Materials and Methods

### 2.1. Study Design

This phenomenological qualitative study aimed to obtain insights into the essence of the participants’ experiences by vividly describing the experiences of nurses with three-shift schedules carrying out clinical work while pregnant in detail from one-on-one, in-depth interviews.

### 2.2. Sampling and Participants

The study participants were nurses working at a tertiary hospital who had been pregnant in the past 3 years while carrying out clinical work. Twelve individuals who volunteered to share their experiences after hearing about the aims of the study and the interview method participated in the interview (Table 1).

### 2.3. Interviews

In-depth interviews were conducted by the two female authors one-on-one at a time the participant preferred and in a quiet location where they felt comfortable. The interviews lasted from 40 min to 90 min, and each participant was interviewed once. All of the interviews were audio-recorded by a digital recorder. A semi-structured interview guide was used to construct the interview questions through discussion among researchers and a literature review. The participants were notified of the research topic and research questions before the interview, giving them ample time to consider the research topic. The main question for the interview was: “How was your work experience as a nurse in the hospital while being pregnant?” Other questions included: “What is the meaning of pregnancy for you?”, “What changes did you experience after pregnancy while working as a nurse?”, and “How was the experience of the process of notifying superiors about your pregnancy?”

### 2.4. Data Collection

For data collection, recruitment advertisements for research participants were posted on online communities where nurses are active. Participants were recruited through snowball sampling. Data collection took place from 13 January to 7 June 2020. Participants were recruited from various regions including Seoul, Daejeon, North Chungcheong Province, and Busan. As data collection and data analysis were conducted at the same time, data collection was finished when no new information was generated from the interviews; therefore, data from 12 participants were finally included. In phenomenology research, it is important to be mindful that communication takes place through the medium of conversation. For that reason, participants’ body language (e.g., movements, postures, and gestures) was recorded in interview notes both when they were speaking and during periods of silence. The primary authors ensured that the interpretation and meaning of the interview were clarified prior to completing the interview by using a summary approach, wherein the interviewer recounted the main themes to the participant. The participant was then asked to either confirm these themes or to guide the primary authors in clarifying the interpretation of the conversation.

### 2.5. Data Analysis

The data transcribed by two assistant researchers were analyzed through the following process utilizing the qualitative data analysis method suggested by Colaizzi [19]. First, the recorded interview content with the participants was listened to multiple times and transcribed exactly. In order to get a sense of the transcribed content, transcripts were read repeatedly by the researchers to understand and interpret the participants’ statements. Meaningful statements were then extracted from phrases and sentences that dealt with the phenomena studied, and the meaningful statements were restated in more general formats. The meanings extracted from the meaningful statements and reformatted statements were organized into themes, thematic clusters, and categories. The themes were then linked to the phenomena of interest, resulting in a final narration in complete, clear sentences. This data analysis process was conducted by three researchers simultaneously, and the selected sentences and phrases and the process and results of categorization were reviewed. The co-first authors coded and analyzed the data separately and the third author supervised the process. The researchers held in-depth discussions until they reached consensus. Participant checking, involving six participants, was conducted to confirm whether the themes extracted from the data reflected the participants’ experiences well.

### 2.6. Preunderstanding

All of the co-authors who participated in this study are women and have knowledge and experience with qualitative research methods. The co-authors attended a course on qualitative research methods during their graduate program and received training on interview methods and qualitative research methods. Before conducting this study, the co-authors participated in a seminar titled “Process and Procedures of Qualitative Research” to learn about coding and categorization of qualitative data, classification, interview methods, and manuscript writing.

### 2.7. Ethical Considerations

This study was approved by the Institutional Review Board (IRB) of the university with which the authors were affiliated before data collection (IRB No. 2019-355-01). This study was conducted in accordance with the principles of the Declaration of Helsinki and the guidelines provided by the IRB. The written consent form stated the purpose of the study, the data collection method, the duration of the interview and that it would be recorded, the voluntary nature of study participation, the right to refuse participation, and confidentiality, and the form was explained to the participants again before the beginning of the interview. Moreover, it was explained that they could discontinue their participation even during the interview and that there would be no disadvantage as a result of discontinuation. A transportation fee was provided to participants as compensation for participation. The interview recordings and transcripts were coded so that participants could be identified and given separate individual numbers for management.

When the interviews were transcribed, data were differentiated using the researcher’s name and number rather than participants’ personal information. In order to maintain confidentiality and to ensure privacy, participant identification numbers were used when the results were described. The text data were stored in a locked cabinet and will be shredded and discarded after the study is concluded.

## 3. Results

This study analyzed the clinical work experience of pregnant nurses using the methods proposed by Colaizzi [19]. In total, 192 meaningful statements were extracted from the original data collected from 12 research participants and yielded 87 formulated meanings. Based on these, 25 themes, 12 theme clusters, and 6 categories were identified (Table 2).

### 3.1. Category 1: Enduring Alone

This category contains two theme clusters: “emotional changes” and “physical changes.” Nurses who experienced clinical work during pregnancy felt various types of pain due to the emotional and physical changes accompanying pregnancy and endured the period during which they had to work while being pregnant.

#### 3.1.1. Theme 1: Emotional Changes

This theme cluster is composed of four themes: “reading the room,” “times of despair and anxiety,” “feeling sorry about being a burden to colleagues and the organization,” and “pity towards myself for feeling the burden.” The participants discussed the emotional changes and difficulties they experienced due to pregnancy. These emotional changes included feelings toward colleagues and the organization as a result of personal changes and situational changes due to pregnancy.

“Well, it’s only right not to ask a pregnant person to work a night shift. I secretly wished they would exempt me, but I had to read everyone’s reactions. Yeah, I worked like that.”

(Nurse 7)

“I just tried my best to endure. I thought of it like that. I think my anxiety was very severe.”

(Nurse 2)

“The night shifts that I was working, others would need to cover them. My shifts would have to be divided among others…So I felt bad…These things were good for me, but I also felt sorry. Ultimately, would I be able to work again? Should I work? (sigh) I kept having these thoughts because I had to continue working while burdening colleagues.”

(Nurse 4)

“When I was pregnant, well, I could have quit, but I wanted to tell my child that I really lived to the fullest before I met you…(sobs)”

(Nurse 4)

#### 3.1.2. Theme 2: Physical Changes

This theme cluster is composed of three themes: “chronic fatigue, “sleep disorders,” and “different activity level from prepregnancy.” Participants discussed various difficulties and pain from physical reactions that changed with pregnancy.

“There were times that I felt great and light (before pregnancy), but when I was pregnant, I was almost always tired, and my body felt heavy. In this situation, I had to go to work constantly, so there was a lot of pressure. I just endured until my next day off.”

(Nurse 3)

“I was constantly tired. I binge-slept on days off.”

(Nurse 7)

“It was especially difficult during night shifts. For me, even before the pregnancy, I had a hard time changing my sleep pattern quickly for shift work. Sometimes I couldn’t sleep at all even if I had a day shift the next day, so I would take sleeping pills or sleep-inducing pills. But after becoming pregnant, it was difficult to take such drugs…”

(Nurse 3)

“For me, it was difficult towards the end (of the pregnancy) when I had to crouch down like when I had to empty a patient’s drainage tubes. And nurses are just very busy to begin with. I had to run around, but because my belly got bigger, it became harder to catch my breath.”

(Nurse 9)

“I was constantly tired, and I always wanted to sleep. So ever since I got pregnant, I didn’t really spend time with anyone. I would go to sleep right after my shift. I think I just slept. Continuously. I had this weird sleepiness and fatigue.”

(Nurse 5)

“My belly was so huge yet people would ask me to do something as if it was no big deal. I would feel a bit hesitant because it was a lot for me.”

(Nurse 6)

### 3.2. Category 2: Organizational Characteristics of Nursing

This category included two theme clusters: “strict organizational culture” and “manager’s attitude.” The nurses who participated in this study discussed their experiences and the effects of the organizational culture in their workplace on clinical work during their pregnancy.

#### 3.2.1. Theme 1: Strict Organizational Culture

This theme cluster is composed of two themes: “culture that ignores difficulties during pregnancy” and “implicitly existing turn-taking for pregnancies.” While sharing their experiences, participants confirmed the strictness of the organizational culture and environment throughout their pregnancy.

“Difficulties that nurses feel are ignored and nurses are taken for granted. They think their work is just about running the wards without trouble and running the organization. They don’t think to look into how difficult it is for people and what kind of work there is. No matter what, just running the three shifts without trouble. Just prevent medical accidents, nurses accidentally giving the wrong medication, or various other accidents. That’s all they care about.”

(Nurse 3)

“Those with more years of experience say that there was no consideration back in the day. They just did it, unconditionally. They compare the past to now and ask, ‘Why should we do it differently? When I worked in such difficult conditions in the past? So, they think these considerations are out of the ordinary. Because things have improved these days. But things should be improving. They just say it wasn’t like that for them.”

(Nurse 6)

“I unconsciously tend to think that those with more years of experience should get pregnant first. Among the married nurses, there was one who had 3 more years of experience, but someone who didn’t have as much experience got pregnant first. No one said anything in front of the pregnant nurse, but behind her back, they would say, ‘Why would she get pregnant first?’ I also heard the head nurse say ‘Sigh…the one with more experience should have gotten pregnant first.’ But she could have tried but failed. You can’t get pregnant just because you want to.”

(Nurse 8)

#### 3.2.2. Theme 2: Manager’s Attitude

This theme cluster is composed of two themes: “manager’s negative reaction towards pregnancy” and “managers who do not care about maternity protection policies.” Participants discussed the negative reactions that they experienced when notifying managers about their pregnancy and working and their lack of knowledge or indifference towards maternity protection policies.

“The manager’s face turned cold. S/he opened their eyes wide and sighed as if to say they are at a loss for words. They said it isn’t nice to say this, but did you really have to (get pregnant) in this situation?”

(Nurse 3)

“I was between 6 and 7 weeks pregnant when I told the manager because there was the issue of night shifts and all. But the manager said, ‘Listen to the heartbeat more, give it more time. When the pregnancy is more established, come back with a diagnosis slip.’ Rather than congratulating, it was very stiff, very business-like. I felt so hurt and let down. I thought about whether I should keep working or not.”

(Nurse 4)

“The division head doesn’t really know anything and doesn’t care. S/he is constantly busy too. So, I felt that s/he just pretended to handle it. That’s how I felt.”

(Nurse 4)

### 3.3. Category 3: Risky Work Environment

This category includes two theme clusters: “lack of maternity protection work guidelines” and “overly busy work.” Nurses stated that their work environment was very risky to be in while being pregnant, but that there were no guidelines that could protect pregnant nurses.

#### 3.3.1. Theme 1: Lack of Maternity Protection Work Guidelines

This theme cluster contains one theme: “protecting myself in a risky environment.” Nurses indicated that they worked in situations where they were repeatedly exposed to violence, verbal abuse, radiation, physical risks, chemical compounds, and infectious diseases while caring for patients, and that there were no workplace guidelines that protected them. As a result, they were left to take precautions on their own.

“There was a dispute about a tuberculosis patient. Whether they would make us (pregnant nurses) see tuberculosis patients, that became a bit of an issue. Nurses who already had been pregnant and given birth had their own ideas, like ‘You’re not going to see tuberculosis patients just because you’re pregnant? I have a small child at home’…So if there were to be clear standards, there wouldn’t be such controversies. That’s what I think.”

(Nurse 9)

“I am in the intensive care unit, so sometimes the X-ray technician comes in to take X-rays. We need to wear lead aprons. But I can’t be like, ‘Because I’m pregnant I should wear the lead apron first.’ We need to position the child (patient) and hold onto the patient, so I can’t just jump to action to wear the lead apron. I just wait until the very last minute until the nurse next to me or a younger nurse says, ‘you should wear the apron.’ Only then do I wear the apron.”

(Nurse 11)

#### 3.3.2. Theme 2: Overly Busy Work

This theme cluster contains two themes: “work that does not let you rest even for a little bit” and “not recognizing the fetus during work.” Even when they were pregnant, nurses described an extremely busy workflow that did not allow them to rest and even made them temporarily forget about their pregnancy.

“The situation at the labor and delivery ward is like that. Sometimes deliveries come in all at the same time, and sometimes there are no patients at all. So when there are no patients, they just cut the staffing for the shift. Then I need to rest unexpectedly. And then when deliveries come in at once, we take in 4, 5 patients in a row in one shift. In those cases, there’s no time to eat or go to the bathroom. We just keep tending to patients.”

(Nurse 12)

“A lot of times I would forget that I was pregnant at work. Because work was too busy. Because we work in a hospital, there are many emergencies. Like I said, when a patient was in arrest in front of me, I couldn’t think of the fact that I was pregnant. I didn’t think about myself and just attended to the situation.”

(Nurse 3)

### 3.4. Category 4: Strengths that Sustain Work during Pregnancy

This category includes two theme clusters: “support and consideration from colleagues in the same department” and “my occupation that I cannot give up on.” Although there are many difficulties carrying out clinical duties while being pregnant, nurses spoke about what helped them continue working while they were pregnant.

#### 3.4.1. Theme 1: Support and Consideration from Colleagues in the Same Department

This theme cluster contains two themes: “managers providing information about maternity protection and support” and “colleague nurses taking on duties and being considerate.” Despite the difficulties in carrying out clinical work while being pregnant, nurses described how the help and support from other nurses and managers in the same department allowed them to keep working.

“The head nurse first told me that I could take 2 h each day (off) and count that as annual leave. After that I gained some more annual leave days, so when I have more annual leave days, I can use that instead of sick leave. So, the head nurse combined all the hours and made them into one annual leave day.”

(Nurse 5)

“Nurses who had already been pregnant on the job and given birth were very understanding. When I had morning sickness, they told me to eat this and that and told me to go into an empty treatment room to rest.”

(Nurse 2)

#### 3.4.2. Theme 2: My Occupation That I Cannot Give up on

This theme cluster includes two themes: “pride as a nurse” and “fear of career disruption.” The nurses took pride in their occupation and did not want their careers to be disrupted due to their pregnancy. These factors became strengths that kept them going without quitting.

“While I was pregnant, listening to what others were saying, I realized that my workplace was quite good, and my occupation was really quite decent. Before I got pregnant and I got married, I didn’t have any of these thoughts. I was just totally occupied by how difficult the job was. After I became pregnant, I started seeing the benefits I gained from the hospital. I felt the benefits. So, I gained love for my workplace and satisfaction for my job.”

(Nurse 5)

“I’m now in my sixth or seventh year on the job. If I think about quitting now, I feel it’s such a waste. I don’t think I can go to another hospital, another teaching hospital.”

(Nurse 7)

### 3.5. Category 5: Growth as a Nurse

This category is composed of two theme clusters: “trust from patients and patients’ families” and “an increased sense of responsibility and empathy.” The nurses had a difficult time carrying out their clinical duties while they were pregnant, but stated that their pregnancy became an opportunity to gain trust from patients and patients’ families and served as an impetus for personal growth.

#### 3.5.1. Theme 1: Trust from Patients and Patients’ Families

This theme cluster has one theme: “trust that high quality care will be provided.” When patients and patients’ family members see that a nurse is providing care while being pregnant, they trust that the nurse will provide care with great responsibility and empathy. Nurses stated that this helped building rapport with patients and patients’ families.

“The families of patients gave me more trust, thinking that I was a pregnant nurse in a pediatric unit, so I must take care of my patients very well. They looked back on their pregnancies through mine.”

(Nurse 11)

#### 3.5.2. Theme 2: Increased Sense of Responsibility and Empathy

This theme cluster includes two themes: “deeper responsibility and understanding for patients and patients’ families” and “ability to understand and care about other nurses”. Through the experience of pregnancy, nurses reported that their ability to empathize with patients and patients’ families increased so that they could provide the best care possible. Nurses could also provide more understanding and care to other nurses when they became pregnant.

“I think I feel more responsibility. You need to be responsible to provide care to patients. Because now I have a child, I have more responsibilities, so when I interact with patients, I take more responsibility and think I should provide more detailed explanations, and so on.”

(Nurse 6)

“I did not know that morning sickness is that difficult. But because I had a very severe case of morning sickness, it helps. I can empathize.”

(Nurse 2)

### 3.6. Category 6: Methods to Protect Pregnant Nurses

This category includes two theme clusters: “development and implementation of maternity protection policies appropriate for the nursing occupation” and “improvement in social perceptions of pregnant nurses.” The maternity protection policies currently in place in South Korea are not applicable to nurses who work shifts, and under these circumstances, nurses pointed out problems such as situations where their pregnancies adversely impacted their colleagues.

#### 3.6.1. Theme 1: Development and Implementation of Maternity Protection Policies Appropriate for the Nursing Occupation

This theme cluster includes two themes: “maternity protection policies that are hard to apply to the nursing occupation” and “urgent need to increase nursing personnel.” The current reduced work hour policy is not applicable for nurses who work shifts, so nurses are given alternative leave days instead. Since pregnant nurses cannot take night shifts, their colleagues must take more night shifts. Pregnant nurses expressed that they needed to work, but constantly felt sorry and uncomfortable. Nurses suggested that nursing personnel planning and supplementation should take into consideration the leave periods for pregnancy and birth for women in their reproductive years.

“I cannot even imagine. If I work 2 h less each day, other people need to make up for that.”

(Nurse 1)

“Because we are all human, when we don’t have enough people and the amount of work I need to do increases, it becomes stressful. We become more sensitive to others, less cordial with colleagues, and less kind to patients. We could not help that, right? So, I sometimes think, even if I don’t get paid as much, I wish there were more people.”

(Nurse 5)

#### 3.6.2. Theme 2: Improvement in Social Perceptions of Pregnant Nurses

This theme cluster includes two themes: “improvements in recognition of the need to protect pregnant nurses early in their pregnancy” and “improvement in managers’ perceptions of pregnant nurses.” Because early pregnancy is not apparent to colleagues, nurses can inadvertently be exposed to inappropriate risks. Nurses also emphasized the need for improvements in managers’ attitudes and perceptions of pregnant nurses as a burden to the department.

“I think that the maternity uniform for pregnant nurses should be given early on, because patients’ family members should know that we are pregnant. Then they can be more careful.”

(Nurse 7)

“A junior nurse I worked with was a bit frail. She had low blood pressure, and she was very skinny. She felt dizzy and was lying down in the treatment room. The head nurse saw that and got very angry that she was behaving like that in the workplace. After that, it was so obvious that that nurse was not liked. After that … she submitted a doctor’s slip, but the manager told her she was being rude. Doing that after taking time off. It’s like that. … First of all, the manager shouldn’t create such environment.”

(Nurse 3)

## 4. Discussion

This study applied a phenomenological approach to determine the essence of the clinical work experience during pregnancy reported by nurses in order to understand their experiences in depth.

The nurses who participated in this study worked each day with the mindset that they needed to endure until giving birth due to the physical and mental difficulties that were exacerbated by conducting clinical work while pregnant. “Enduring alone” was a theme that was extracted from all participants.

Once pregnant, women must adjust to various physical changes. Severe nausea and vomiting, which are mostly experienced early in pregnancy, and depression, which is experienced throughout pregnancy, negatively affect a pregnant women’s daily life and quality of life [20,21]. Since this situation is harmful not only for pregnant women, but also for their fetuses, various countries have implemented maternity protection policies [22]. However, the participants in this study tried to appease their ward managers and felt sorry about burdening colleagues with extra night shifts as soon as they realized they were pregnant. In recent years, pregnant nurses in South Korea have mostly been exempted from night shifts [5], and the burden is shifted to colleagues instead. Nurses then think that their pregnancy is burdensome for their colleagues and the department.

This result aligns closely with the finding of a previous study that nurses had to take on the tasks that could not be performed by pregnant nurses, who felt sorry towards their colleagues [6]. In this study, feeling sorry towards colleagues made it difficult for pregnant nurses to voice their concerns about being exposed to risky work environments and the physical and mental difficulties they experience. This problem occurs because the workload of nurses is high and there is a shortage of nursing personnel [7,8]; therefore, this issue will be difficult to solve without personnel improvements.

Many nurses who participated in the study stated that their managers perceived their exemption from night shifts and maternity leave as headaches that caused personnel loss. In this organizational atmosphere, pregnant nurses felt that maintaining their pregnancy was risky due to their extreme workload and physical limitations, but they were not able to discuss these risks with managers. Ward managers have an important influence on nurses’ job stress and turnover intention [23]. The nurses in this study also reported that they seriously considered quitting due to the ward managers’ negative attitudes and reactions toward pregnant nurses. Nurse turnover ultimately has a negative impact on organizational outcomes through a lack of personnel, the increased workload of the remaining nurses, and negative patient health outcomes [24,25]. As the rate of turnover among South Korean nurses is increasing each year [26], managers’ negative perceptions and attitudes toward pregnant nurses should be improved immediately. Ward managers, who have the responsibility to operate the wards (including nurse staffing management), may also experience high levels of stress when operating a ward with limited personnel. However, in order to ensure that this stress can be dealt with rationally, rather than projected onto pregnant nurses, hospitals should prepare a supplementary workforce system to address the personnel implications of pregnancy. In addition, hospitals should implement leadership training programs for nurse managers, with the goal of helping them to change their negative thoughts and feelings toward pregnant nurses. Initiatives of this type may slowly change the organizational atmosphere of nursing.

The pregnant nurses who participated in this study forgot that they were pregnant because they were so busy during work and sometimes bumped their stomachs on beds. They did not have enough time to go to the bathroom. Nurses also reported they were extremely anxious about the safety of their fetuses because there were no work guidelines to protect pregnant women and their fetuses in high-risk situations, such as handling dangerous drugs (e.g., those used for chemotherapy), treating patients with infectious diseases, or being exposed to radiation. South Korea’s maternity protection policies recommend that pregnant workers should be exempted from night shifts, have reduced hours, and be transferred out of high-risk departments [5]. However, there are no policies that are appropriate to nursing practice, and this study found that some workplaces still have pregnant nurses work night shifts after providing consent.

Women’s social role is expanding and the proportion of working women is increasing in South Korea, but the female participation rate in the economy is relatively low compared to that in other countries [27]. The birth rate in South Korea is especially low, severe enough to be classified as a country with an extremely low birth rate [27]. The sociologist Skocpol [28] claimed that issues around women’s pregnancy should be the focus of policymaking, since these issues are a public good socially and policy-wise. From this perspective, pregnancy and birth experienced by nurses, who are among the core personnel in providing health care services, should not be recognized as issues that individuals endure in isolation. Pregnant nurses should instead receive social protection and their work environment should be improved in an active manner.

Another finding that should be highlighted from this study is that the nurses continued to work due to the pride they had in their job and their fears of career disruption despite the physical and mental hardships they experienced due to pregnancy. Colleague nurses’ consideration and support became the driving force for pregnant nurses to keep working without quitting. According to a survey, more than 96% of nurses in South Korea are women, and the personnel crisis due to the lack of experienced nurses is very severe [1,8]. Acquiring and maintaining adequate nursing personnel is a core element of maintaining the quality of health care services, and experienced nurses perform an especially important role in providing quality nursing care. However, many experienced nurses have trouble maintaining their career due to disruptions resulting from pregnancy, birth, and child-raising [1,8]. This result reflects the situation of nurses, who often find it difficult to work and have a family at the same time. In South Korea, the government has implemented various maternity protection policies (support for pregnancy, childbirth, and lactation; prohibition of harmful working environments; prenatal break; childcare leave; and women’s employment promotion incentives) [29,30,31]. However pregnant nurses working in the field had very limited awareness of these policies, which showed that these policies were not being implemented well. Therefore, the government needs to actively follow up and investigate whether policies are being implemented in actual work settings. The appropriate utilization of maternity protection policies is the only way to maintain a healthy society and to enable female nurses to continue their careers.

Growth as a nurse, found in the fifth theme cluster, was a very meaningful result. The nurses in this study stated that experiencing pregnancy increased the sense of responsibility that they felt in caring for patients. A participant who worked in the pediatric intensive care units and pediatric wards found that her pregnancy facilitated rapport since she felt that patients’ families trusted her more. When nurses were able to successfully navigate their pregnancy due to active support from managers, nurses’ loyalty for the nursing organization and their affection for the hospital increased. They also became able to understand the pregnancy experiences of other nurses more deeply. Nurses experienced many difficulties in their work due to pregnancy, but these difficulties also became opportunities to grow into more mature nurses. This finding was similar to previous reports that organizations and hospitals that actively implemented maternity protection policies had positive effects such as improvements in public image, increases in productivity, reductions in the turnover rate, and increases in intention to work [29,30]. If experienced nurses who have been pregnant can remain in the field and plan to improve the quality of nursing, positive effects on patient health outcomes and hospital performance can be expected.

Nonetheless, hospital administrators may perceive initiatives to strengthen maternity protection policies, including those that protect pregnant nurses, as cost increases [31]. In contrast with the intention of the government’s policy to support work and family at the same time, some organizations might avoid employing pregnant nurses. Therefore, the government should take greater social responsibility for maternity protection to reduce the burden faced by hospitals in implementing maternity protection policies. Hospitals should also pay attention to previous cases where the implementation and utilization of maternity protection policies contributed to strengthened organizational competency and a reduced staff turnover rate [29,30]. Based on these considerations, efforts should be made to develop and implement birth encouragement policies that are applicable to nurses’ work characteristics. Such initiatives will prevent the career disruption of capable experienced nurses due to pregnancy and birth and ultimately contribute to the improvement of health care service delivery.

The participants in this study were found to be affected by national policies and hospital systems. Furthermore, the study was conducted in a regional and acute care clinical setting, which means that the findings have limited generalizability to other countries or other hospital systems, specialties, and work environments. In future studies, it is necessary to conduct additional studies in various regions and fields where nurses work. In addition, several participants shared their experiences of miscarriage during the interviews. We suggest examining this phenomenon in more detail through a mixed-method research design that includes nurses’ experiences of both pregnancy and miscarriage. Despite these limitations, the present study derived the experiences and in-depth meaning of pregnancy during clinical work as a nurse.

## 5. Conclusions

This qualitative study explored the clinical work experiences of nurses who had been pregnant in the past three years with the goal of obtaining insights into the essence of those experiences. Nurses experienced various physical and mental changes and difficulties due to pregnancy, and the clinical environment did not provide sufficient support to pregnant nurses. However, experiences of pregnancy improved the nurses’ responsibility and empathy and became a positive opportunity for nurses to grow.

In order for nurses, who are core health care personnel, to be able to conduct clinical work while being pregnant, practical maternity protection policies and work guidelines should be developed and implemented. Not only would this protect pregnant nurses and their fetuses, but in the long term, these steps are necessary to improve the provision of quality nursing care and to ensure patient safety.

## Figures and Tables

**Table 1:** General characteristics of study participants (N = 12).

ID	Age (Years)	Marital Status	Nursing Unit	Total Clinical Career	Number of Pregnancies	Number of Deliveries
1	41	Married	Medical ward	17 years 10 months	4	2
2	31	Married	Surgical ward	6 years	3	1
3	41	Married	Surgical ward	15 years 10 months	1	1
4	32	Married	Medical ward	5 years 6 months	1	1
5	36	Married	Surgical ward	13 years	1	1
6	39	Married	Surgical ward	14 years	3	2
7	28	Married	Medical ward	7 years	1	1
8	28	Married	Surgical ward	5 years 7 months	1	1
9	29	Married	Medical ward	5 years	2	2
10	33	Married	ICU ^1^	9 years	2	1
11	33	Married	ICU ^1^	11 years 3 months	1	1
12	32	Married	ICU ^1^	10 years 5 months	2	1

^1^ Intensive care unit.

**Table 2:** Categories, theme clusters, and themes of nurses’ clinical experience during pregnancy.

Categories	Theme Clusters	Themes
1. Enduring alone	Emotional changes	Reading the room
		Times of despair and anxiety
		Feeling sorry about being a burden to colleagues and the organization
		Pity towards myself for feeling the burden
	Physical changes	Chronic fatigue
		Sleep disorders
		Different activity level from prepregnancy
2. Organizational characteristics of nursing	Strict organizational culture	Culture that ignores difficulties during pregnancy
		Implicitly existing turn-taking for pregnancies
	Manager’s attitude	Manager’s negative reaction towards pregnancy
		Managers who do not care about maternity protection policies
3. Risky work environment	Lack of maternity protection work guidelines	Protecting myself in a risky environment
	Overly busy work	Work that does not let you rest even for a little bit
		Not recognizing the fetus during work
4. Strengths that sustain work during pregnancy	Support and consideration from colleagues in the same department	Managers providing information about maternity protection and support
		Colleague nurses taking on duties and being considerate
	My occupation that I cannot give up on	Pride as a nurse
		Fear of career disruption
5. Growth as a nurse	Trust from patients and patients’ families	Trust that high quality care will be provided
	An increased sense of responsibility and empathy	Deeper responsibility and understanding for patients and patients’ families
		Ability to understand and care about other nurses
6. Methods to protect pregnant nurses	Development and implementation of maternity protection policies appropriate for the nursing occupation	Maternity protection policies that are hard to apply to the nursing occupation
		Urgent need to increase nursing personnel
	Improvement in social perceptions of pregnant nurses	Improvements in recognition of the need to protect pregnant nurses early in their pregnancy
		Improvement in managers’ perceptions of pregnant nurses

## Data Availability

The data presented in this study are available on request from the corresponding author. The data are not publicly available due to privacy or ethical restrictions.
